# Integration of Umbilical Cord Mesenchymal Stem Cell Application in Hydroxyapatite-Based Scaffolds in the Treatment of Vertebral Bone Defect due to Spondylitis Tuberculosis: A Translational Study

**DOI:** 10.1155/2021/9928379

**Published:** 2021-08-23

**Authors:** Ahmad Jabir Rahyussalim, Ahmad Nugroho, Muhammad Luqman Labib Zufar, Irfan Fathurrahman, Tri Kurniawati

**Affiliations:** ^1^Department of Orthopaedic & Traumatology, Cipto Mangunkusumo General Hospital, Faculty of Medicine Universitas Indonesia, Jakarta, Indonesia; ^2^Stem Cell Medical Technology Integrated Service Unit, Cipto Mangunkusumo General Hospital, Jakarta, Indonesia; ^3^Stem Cells and Tissue Engineering Research Cluster, Indonesian Medical Education and Research Institute (IMERI), Faculty of Medicine Universitas Indonesia, Jakarta, Indonesia

## Abstract

**Background:**

Vertebral bone defect represents one of the most commonly found skeletal problems in the spine. Progressive increase of vertebral involvement of skeletal tuberculosis (TB) is reported as the main cause, especially in developed countries. Conventional spinal fusion using bone graft has been associated with donor-site morbidity and complications. We reported the utilization of umbilical cord mesenchymal stem cells (UC-MSCs) combined with hydroxyapatite (HA) based scaffolds in treating vertebral bone defect due to spondylitis tuberculosis.

**Materials and Methods:**

Three patients with tuberculous spondylitis in the thoracic, thoracolumbar, or lumbar region with vertebral body collapse of more than 50 percent were included. The patient underwent a 2-stage surgical procedure, consisting of debridement, decompression, and posterior stabilization in the first stage followed by anterior fusion using the lumbotomy approach at the second stage. Twenty million UC-MSCs combined with HA granules in 2 cc of saline were transplanted to fill the vertebral bone defect. Postoperative alkaline phosphatase level, quality of life, and radiological healing were evaluated at one-month, three-month, and six-month follow-up.

**Results:**

The initial mean ALP level at one-month follow-up was 48.33 ± 8.50 U/L. This value increased at the three-month follow-up but decreased at the six-month follow-up time, 97 ± 8.19 U/L and 90.33 ± 4.16 U/L, respectively. Bone formation of 50-75% of the defect site with minimal fracture line was found. Increased bone formation comprising 75-100% of the total bone area was reported six months postoperation. A total score of the SF-36 questionnaire showed better progression in all 8 domains during the follow-up with the mean total score at six months of 2912.5 ± 116.67 from all patients.

**Conclusion:**

Umbilical cord mesenchymal stem cells combined with hydroxyapatite-based scaffold utilization represent a prospective alternative therapy for bone formation and regeneration of vertebral bone defect due to spondylitis tuberculosis. Further clinical investigations are needed to evaluate this new alternative.

## 1. Introduction

Vertebral bone defect (VBD) continues to exist as one of the main skeletal problems in the spinal region. This defect—resulting in discontinuation of the spinal column—will disturb the structural functions of the spine and its surrounding tissues. With vertebral fracture due to osteoporotic bones as the major cause of the defect, another important etiology which is in need to be put into consideration is spondylitis tuberculosis [1, 2]. This condition remains to be a major health problem with perilous and life-threatening morbidity and mortality. In addition to the defect, spondylitis tuberculosis is also associated with poor pain control, pathological fracture, structural instability, neurological deficit, and deformity. With severe inflammatory reaction and disease progression, caseation and sclerosis of the bone and tissue may emerge. This further contributes to the inhibition of bone matrix synthesis, increased degradation of the bone, and collapse of the vertebral structures [3–5].

Until these days, debridement, decompression, vertebroplasty, kyphoplasty, and stabilization—in addition to antituberculosis drugs—still become the principal management in treating patients with vertebral bone defect due to spondylitis tuberculosis. With fusion as the principal aim, bone graft is used in the spinal surgery of these cases. Autologous bone graft becomes the standard option in treating the bone defect since it possesses natural osteogenic (cells), osteoinductive (growth factors), and osteoconductive (extracellular matrix) characteristics to promote bone healing, regeneration, and repair [6, 7]. Nevertheless, harvesting the graft in the patient is associated with numerous major drawbacks. Inconsistent quality and quantity of the graft are found to depend on the patient's health condition and age. Besides, there is always a high possibility of donor-site morbidities, such as persistent and prolonged pain at the removal site, scar tissues, deep infection, vascular injuries, and sciatic nerve injury. Further complications will result in gait disturbance and abnormalities [8].

The use of an allograft as a substitute presents the risk of infection transmission, immune rejection, postoperative infection, and refracture. On the other hand, there is unpredictable strength and degradation rate of the synthetic scaffolds depending on the anatomical site and patient's clinical condition [9, 10]. Nevertheless, fusion failure rate of the vertebrae in the defect area remains to be high—roughly 25-40%—even with standardized procedures. Not only the grafts, surgical technique, implant selection, and patients' general conditions (health status, comorbidity) but also habits (tobacco smoking, alcohol consumption, and daily activities) play important roles in the healing process after the performed procedure [11–13]. Therefore, these conditions clarify the need for an alternative and effective modality of treatment regarding bone defect in spondylitis tuberculosis cases. The use of culture-expanded mesenchymal stem cells (MSCs) combined with biomaterial scaffolds has become a significantly attractive choice. This combination may carry out the osteogenic, osteoinductive, and osteoconductive requirement in bone regeneration and healing [13, 14].

The use of bone marrow mesenchymal stem cells (BM-MSCs) remains to be the gold standard in regenerative medicine. However, these cells have a lower potential to proliferate. Besides, its extraction is associated with painful and invasive collection procedures. Before regenerative medicine, larger bone defect was treated using bone grafts either autologous bone grafts or allograft. Iliac crest harvest is considered as the gold standard; however, bone graft method is less preferred because of the lack of osteogenic properties, lack of massive bone source, incomplete bone remodeling, infections, and risk of morbidities at the donor site. Success with alternative method such as mesenchymal stem cells has been reported in many cases [15].

On the other hand, umbilical cord mesenchymal stem cells (UC-MSCs) hold a higher proliferation with greater immunomodulation capacity. A review by Zhuang et al. even shows MSCs as promising therapeutic strategy still shadowed with tumor initiation risk; UC-MSC however shows the highest tumor suppression properties compared to other MSCs and with the lowest tumor promotion effects compared to other types of MSCs [16]. These safety profiles have put UC-MSCs as another promising option to be the precursor of allogeneic MSCs in bone regeneration. These cells originate from fetal tissues and can be isolated from Wharton's jelly. In addition, UC-MSCCs are easier and simpler to collect compared to BM-MSCs [17, 18]. Enhancement using osteoconductive scaffolds may improve and amplify the results regarding bone formation and remodeling. However, contamination and infection issues emerge from the frozen cadaveric materials. Hydroxyapatite (HA) scaffolds have been associated with high effectiveness in the treatment of long bone defect, spinal fusion, and craniofacial fractures. This is due to its high porosity, interconnected structures, and exceptional biocompatibility features. Moreover, this synthetic calcium phosphate-based bioceramic scaffold does not affect MSC characteristics of proliferation, differentiation, and immunomodulation activity [19, 20].

Anterior surgery using lumbotomy approach offers great exposure of the lumbar vertebrae with straight visualization for debridement, decompression, and stabilization. Despite a more complex and complicated procedure with superior anatomic and technical competence requirements, this approach is reported to have better favorable clinical and functional outcomes. Spinal reconstruction of spondylitis tuberculosis, in which it affects the anterior vertebral body predominantly, may become the fundamental indicator for the procedure to perform [21, 22]. Despite that, there are only a small number of studies reporting MSC effectivity combined with synthetic osteoconductive scaffolds in the treatment of vertebral bone defect, especially due to spondylitis tuberculosis. In this report, we discussed three cases of VBD due to spondylitis tuberculosis treated with umbilical cord mesenchymal stem cells integrated within hydroxyapatite scaffolds using lumbotomy approach.

## 2. Materials and Methods

### 2.1. Patients

A prospective second-phase clinical trial was performed between January 2019 and June 2021 in Cipto Mangunkusumo National Referral Hospital (RSCM). This study has been approved by the Ethics Committee of the Faculty of Medicine, Universitas Indonesia (No. 0489/UN2.F1/ETIK/2018).

The inclusion criteria of this study were patients with tuberculous spondylitis in the thoracic, thoracolumbar, and lumbar region with vertebral body collapse of more than 50 percent, evaluated using X-ray on the anteroposterior and lateral projection. Besides, patients have undergone medications with anti-TB regimens for at least two weeks prior to surgical intervention. The exclusion criteria of this study were patients below ten years old or with multilevel vertebral defects. Three patients, who met our criteria and are willing to participate in this study, with written informed consent, were included in this study.

Baseline functional status was assessed in all subjects prior to the study period using clinical examination, radiological evaluation (X-ray and contrast MRI), laboratory evaluation (LED and peripheral blood test), and interview using the questionnaire Short Form (36) Health Survey (SF-36). Written informed consent for publication was obtained from all the patients involved in this study. A copy of the written consent is available for review by the Editor-in-Chief of this journal on request. Characteristics of the subjects are presented in Table 1.

### 2.2. Umbilical Cord Mesenchymal Stem Cells (UC-MSCs)

The UC-MSCs used in this report were isolated in our center from the human umbilical cords (UCs) of women with a healthy pregnancy. These UCs were collected within phosphate-buffered saline (PBS) and 100 U/mL penicillin and 100 *μ*g/mL streptomycin as antibiotic supplementation. Before incubating in the atmosphere of humidified air with 5% CO_2_ in DMEM at 37°C in 25 cm^2^ flasks, UCs were cleaned by flowing PBS over cell culture hood and cut longitudinally into 5 cm^2^ segments. 10% FBS or 10% CBS was put as supplementation for medium conditioning. Further process involved rapid thawing and reculture inside a 25 cm tissue culture (T25) flask. Platelet lysate with 10% concentration in complete medium was used in the procedure.

**Figure 1:**
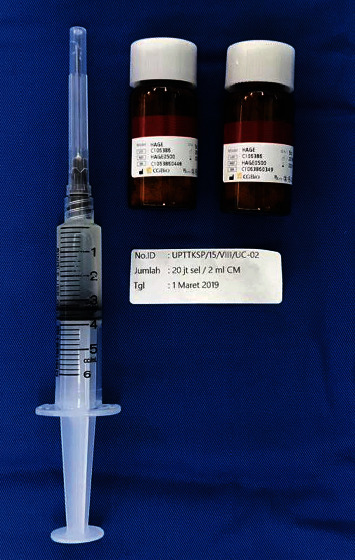
Twenty million umbilical cord mesenchymal stem cells (UC-MSCs) and hydroxyapatite-based scaffolds used in the procedure.

The harvested cells were washed, suspended, counted, and reseeded at 80-90% confluence using trypsin and EDTA 0.05% inside DMEM supplemented with either 10% FBS or 10% CBS. Osteogenic differentiation media (hMSC osteogenic differentiation kit) were put on the UCs for in vitro differentiation. Alizarin red at a concentration of 2% (pH 4.2) was used to detect the calcium deposit for osteogenic differentiation. These samples had been subcultured to the 4th passage and underwent cryopreservation and were stored within liquid nitrogen. Twenty million UC-MSCs were injected towards the vertebral bone defect combined with HA granules in 2 cc of saline following debridement of the affected vertebrae through the lumbotomy approach (Figure 1).

### 2.3. Surgical Procedures

**Figure 2:**
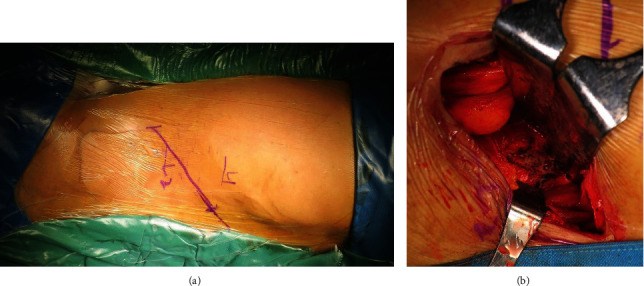
Lumbotomy approach with (a) left lateral skin incision to assess (b) thoracolumbar vertebral level.

**Figure 3:**
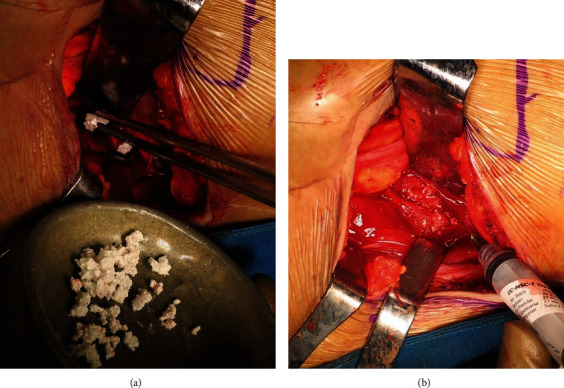
UC-MSCs and hydroxyapatite-based scaffold application on the vertebral bone defect area.

All surgical procedures were performed by one team of three spine surgeons. The patient underwent two stages of surgical procedures. The first stage involved debridement and decompression over the affected site using posterior approach. Posterior stabilization using pedicle screws and Rods system was used to provide mechanical stability. This was followed by second-stage surgery exposing the affected vertebral body using lumbotomy approach. Afterward, 20 million umbilical cord mesenchymal stem cells combined with the granules of hydroxyapatite-based scaffolds (Bongros®-HA, Bioalpha, Seongnam, Korea) in two millimeters of saline were implanted on the bone defect site. Finally, soft tissue closure was performed (Figures 2 and 3).

### 2.4. Evaluation of the Subjects

Postoperatively, the functional outcomes were assessed with the SF-36. The subjects were then followed up at the first month, third month, and sixth month postoperatively for clinical and radiographic evaluations. Bone healing assessments were conducted using the Tiedeman scoring system from the X-ray images to evaluate bone formation of the patients. Besides, the Bridwell scoring system from the CT scan images was used as an interbody fusion grading system. We also performed a laboratory test for alkaline phosphatase in which this enzyme may represent the progression of osteoblast activity and bone regeneration. Lastly, the SF-36 questionnaire was used to evaluate the patients' quality of life quantitatively using a scoring system with 36 questions divided into 8 domains.

## 3. Results

**Figure 4:**
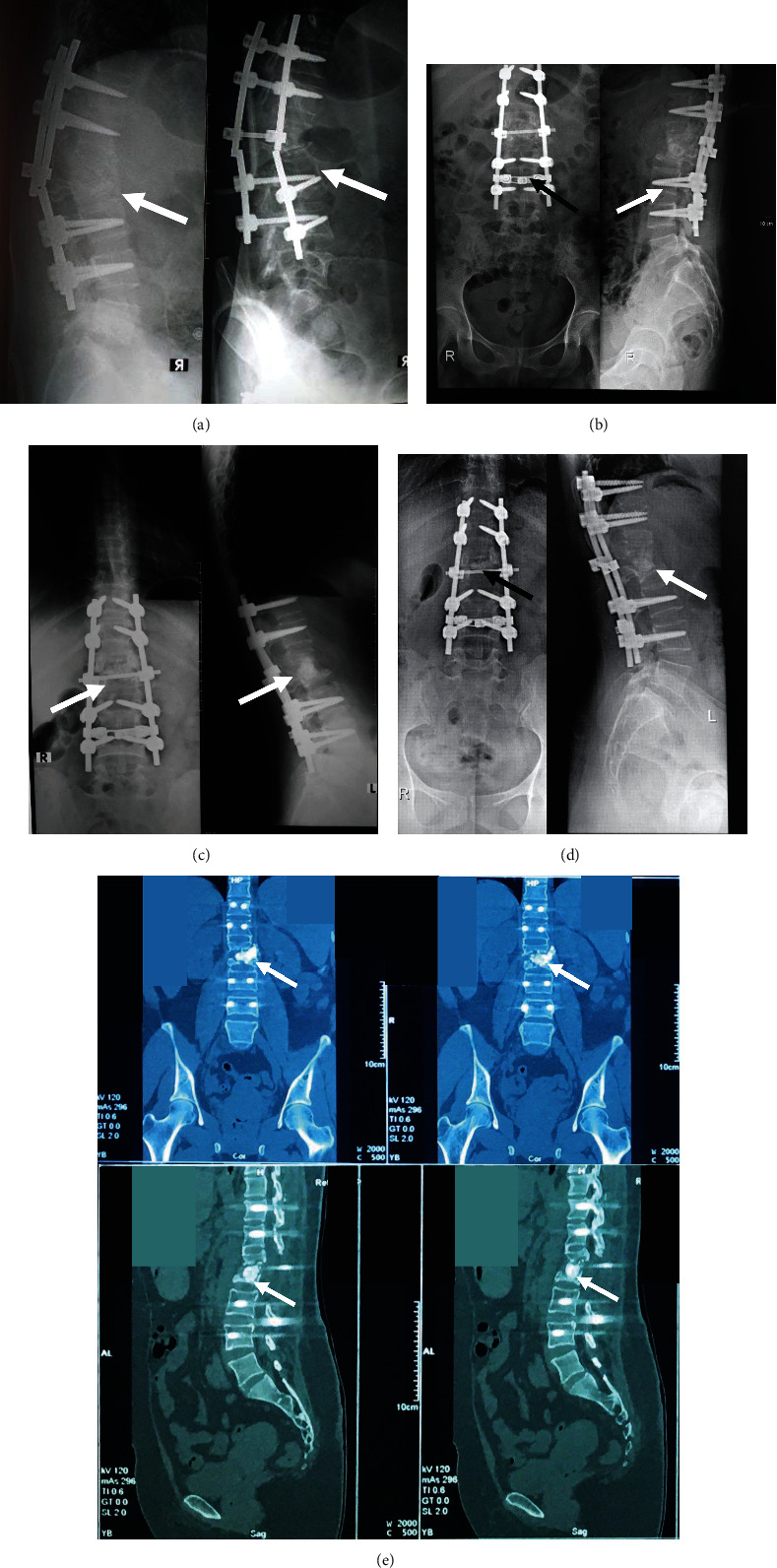
A 29-year-old female (case 1). (a) Preoperative radiography showed large destruction on the corpus vertebrae which causes nonunion and implant failure. (b) Early postoperative radiography showed implant planted posteriorly bridging defected bone with alignment well corrected. (c) Three-month postoperative radiography showed 60% of new bone growth on the defected corpus vertebrae. (d) Six-month postoperative radiography showed 80% of new bone growth on the defected corpus vertebrae. (e) Six-month postoperative CT scan showed 80% of new bone growth filling the bone defect especially on the right posterior lateral. Sites are shown using white and black arrows.

**Figure 5:**
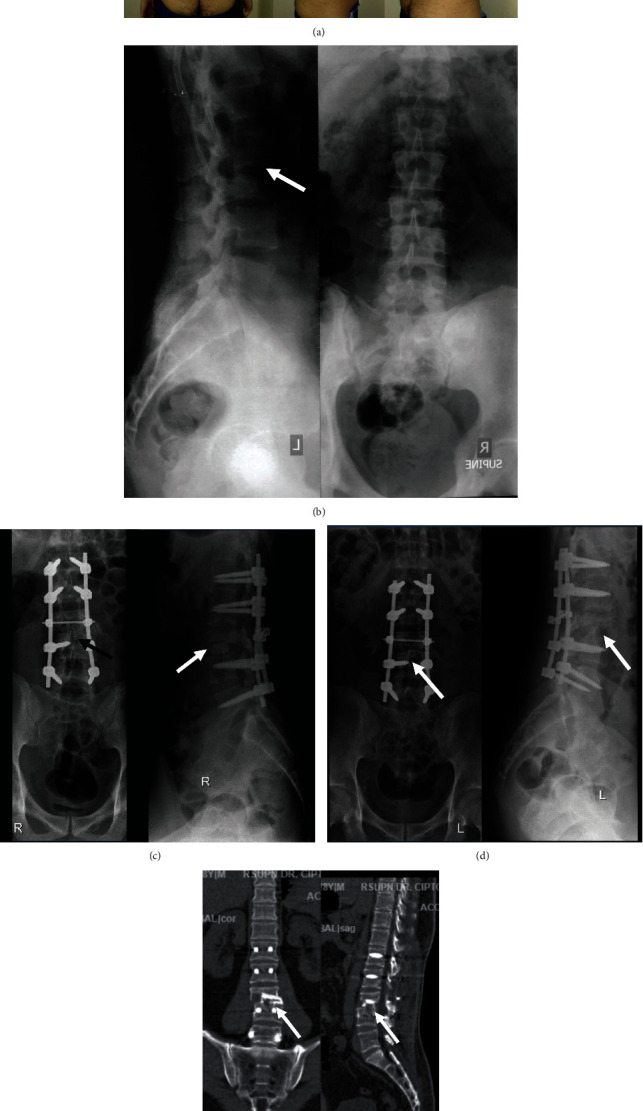
A 27-year-old male (case 2). (a) Preoperative clinical picture showed hyperlordosis. (b) Preoperative radiography showed defect on the anterior column around 55% of the corpus vertebrae. (c) One-month postoperative radiography showed new bone formation that filled around 35% of the corpus vertebrae. (d) Three-month postoperative radiography showed new bone formation that filled around 60% of the corpus vertebrae. (e) Six-month postoperative CT scan showed 60% of new bone formation on the left posterior lateral of the corpus vertebrae. Sites are shown using white and black arrows.

**Figure 6:**
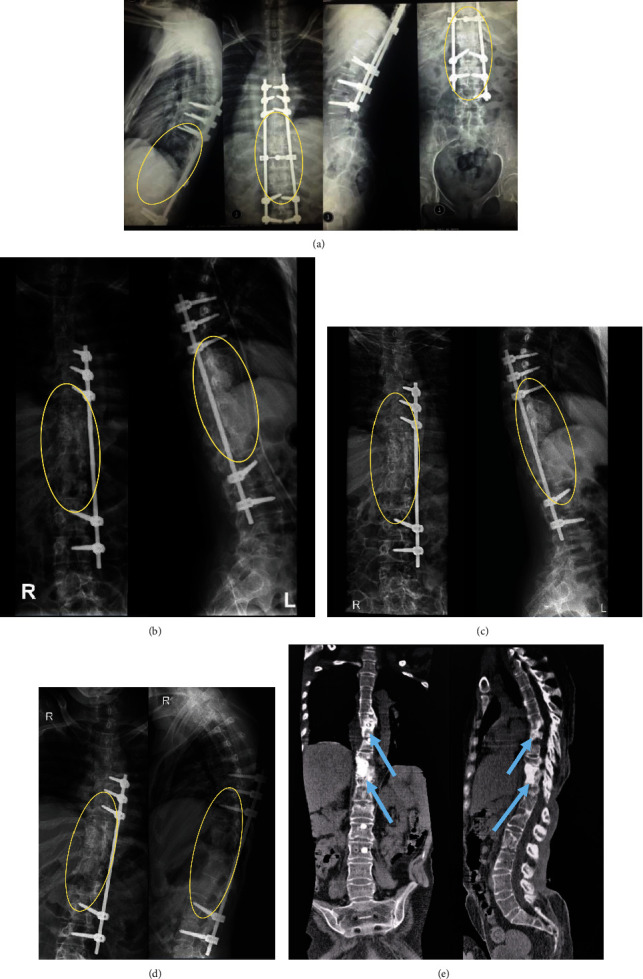
A 30-year-old female (case 3). (a) Preoperative radiography showed defects on multiple corpus vertebrae (T9-12). (b) One-month postoperative radiography showed around 50% of new bone formation along T9-L1, and postoperative radiography also showed instrumentation planted on the left side. (c) Three-month postoperative radiography showed around 65% of new bone formation. (d) Six-month postoperative radiography showed around 85% of new bone formation. (e) Six-month postoperative CT scan showed around 85% of new bone formation. Sites are within yellow circles and pointed using white, black, and blue arrows.

All patients were regularly followed at one month, three months, and six months. No major complications of remarkable bone deformation, spinal cord injury, or infection were observed within the postoperative period in all the cases. Alkaline phosphatase (ALP) levels that reflect osteoblast activities were within normal value at all follow-up points. The initial mean ALP level preoperation was around baseline 41.3 ± 4.16 U/L and at one-month follow-up was 48.33 ± 8.50 U/L. This value increased at the three-month follow-up prior to the decrease towards the lower level at the six-month follow-up time, 97 ± 8.19 U/L and 90.33 ± 4.16 U/L, respectively. A total score of the SF-36 questionnaire evaluating the patients' quality of life showed better progression in every follow-up point with the mean total score at the sixth month of 2912.5 ± 116.67 from all the three patients. These better advancements were observed in all 8 domains of the SF-36 questionnaire. Besides, at the six-month follow-up time, a plain radiograph showed a Tiedeman score of 3-4, and a Bridwell score of grade I-II was reported, showing bone formation throughout 75-100% of total bone volume. In addition, to the diminished fracture line, remodeling over the two cortices, partial to complete cortical fusion, and existing trabecular continuity were also established at this time (Figures 4–6 and Table 2).

## 4. Discussion

Bone defect, especially that affecting vertebrae, presents as one of the most commonly found and challenging orthopedic disorders. This condition mostly occurs in the vertebral compression fractures due to osteoporosis, comminuted fractures due to trauma, malignant metastases of osteolytic tumor, and vertebral hemangioma. Besides, other pathological processes of endocrine and metabolic disorders (such as hyperparathyroidism and Paget disease), inflammatory reactions, and infections may also result in further disruption of vertebral structural integrity [1, 2, 6]. Although conservative treatment may allow satisfactory bone healing in some cases, surgical management is indispensably essential, specifically in the severe cases of critical-sized or large bony defect and in vertebral osteomyelitis. Alternative treatment strategy and approach using different bioartificial graft substitutes and materials need to be developed and discovered since the standard procedure using autologous bone grafts was associated with major drawbacks [2, 5, 6]. In this study, we evaluated the outcome of umbilical cord mesenchymal stem cell application combined with hydroxyapatite-based scaffolds (Bongros®) in the treatment of vertebral bone defect due to spondylitis tuberculosis.

Our study reported that patients had already walked without any pain at three months postoperation. This is well shown in Table 2 that there are significant improvements in patients' SF-36 preoperative score, which improved from 736.67 ± 240.90 to 2340 ± 650.54 at three months postoperation follow-up [23]. Besides, at three-month follow-up time, plain radiograph showed a Tiedemann score of 2-3, representing that there was already 50-75% of bone formation at the defect site with minimal appearance of the fracture line. Grades II and III—based on the Bridwell interbody fusion grading system—were also reported in the CT scan images at three-month follow-up time. These grades indicated the presence of partial fusion with the cortical union and partial trabecular incorporation. Further plain radiograph follow-up at six months postoperation showed increased bone formation comprising 75-100% of total bone formation with minimal to diminished fracture line and remodeling over the two cortices. Partial to complete cortical fusion and trabecular continuity were also established at six-month follow-up time. New bone formation is in line with the elevated ALP level found in our patients as more osteoblast activities thrive in an environment favorable for mineralization of bone growth, resulting in higher ALP levels. A significant amount of ALP level increase was found on three-month postoperation follow-up (Table 2). High ALP level showed directly after three-month follow-up when new bone formation reached 60-65% of the corpus vertebrae (Figures 4–6) [24]. This is in line with the bone remodeling process and the property of osteoblast during bone formation, which started with the differentiation of the MSC population that actively proliferates in the initial stage of osteogenesis. As MSCs commit to becoming osteoblasts, proliferation starts to decrease as osteoblast expresses osteogenic markers such as the ALP to then enter the mineralization phase [25]. Osteoblasts stay around three to four months and this is why we found the peak of ALP level in our patient during the three-month follow-up. This bone remodeling cycle occurs in the span of 120-200 days in which again we found the ALP level in our patients started to drop during the six-month follow-up [25, 26].

Furthermore, there were no remarkable bone deformations, spinal cord injuries, or infections reported in our patients. Our observation was consistent with previous studies showing no sign of any neoplasm formation and development from the defect area embedded with MSCs [6, 27]. This might demonstrate that our approaches in MSC transplantation and handling procedure were safe.

Previously, there are no studies reporting hUC-MSC application in combination with hydroxyapatite-based scaffold effect in treating vertebral bone defects in humans. Most of the already existing studies were carried out in animals and long bones. Cei et al. reported the use of bone particles combined with hUC-MSCs in the weaned rabbit regarding the repair of lumbar vertebral bone defects. Three groups were composed of the control group, bovine bone collagen powder (BBCP) group, and BBCP+hUC-MSC group. The BBCP+hUC-MSC group showed the best result regarding the healing of bone defect compared to other groups. Gross morphology revealed the full bone regeneration on the defect area and restored spinous process structure. This was supported by the result of X-ray lateral scanning showing newly woven bone formation after 3 months of surgery and immunohistochemical analysis showing 100% of positive RuX2 and OPN staining [28]. The result of this report was consistent with the study by Vanecek et al. in which significant bone formation was found in the rat group treated by 5 million MSCs within HA bone scaffold compared to rats treated only by HA scaffold or 0.5 million MSCs [6].

Regarding gene-modified adult stem cell utilization, labeled porcine adipose tissue-derived stem cells (ASCs) with lentiubiquitin promoter-driven firefly luciferase and GFP (LUBFG) overexpressing recombinant human bone morphogenetic protein (rhBMP)-6 were used by Sheyn et al. in treating vertebral bone defect. Better results were found in the treatment group using 1 million pASC-LUBFG cells deposited within 10 *μ*L of fibrin gel (FG; Tisseel kit, Baxter, Vienna, Austria). These were confirmed by longitudinal micro-CT-based quantitative analysis of 100% bone regeneration and significantly higher rate of connectivity density at 4, 6, and 12 weeks postsurgery compared to the fibrin gel-only group [29]. Other studies using bioactive scaffold of sintered poly lactic-co-glycolic acid (PLGA) microsphere, mineralized and demineralized allograft with hyaluronic acid (AFT Bone Void Filler™) combination, and calcium phosphate cement (CPC) augmentation also reported similar results [30–32]. Furthermore, the findings of the current study were consistent with our previously conducted study in which tuberculous spondylitis rabbits were treated by 6 × 10^6^ BM-MSCs transplanted within 150 mg hydroxyapatite. Significant greater ossification score of more than 30 was reported compared to the controls [2].

The osteogenic potential in bone tissue regeneration has put MSCs to be prospective therapeutic tools in the case of diseases affecting bone tissues. Besides, it also has immunomodulatory properties and can be obtained, cultured, and manufactured in large amounts. Trophic factors are secreted by MSCs to promote and accelerate new bone formation and remodeling, including insulin-like growth factor-1 (IGF-1), vascular endothelial growth factor (VEGF), epidermal growth factor (EGF), and transforming growth factor-beta 1 (TGF-*β*1) [33, 34]. It may have an impact and regulate cell migration, proliferation, survival, and differentiation into osteoblasts or other bone lineages with enhanced angiogenesis. Besides, its anti-inflammatory and immunomodulatory activity may impede, suppress, and modulate immune cell activation and proliferation (T and B lymphocytes, macrophages, natural killer cells, and dendritic cells) [9, 35, 36]. Regarding bone formation by the transplanted MSCs, BMP, TGF beta, WNt signaling, notch signaling, hypoxia, dynamic loading with Runx2, Osterix, Sox5, Sox 6, and Sox 9 exert direct influence. Besides, there are also indirect effects that help to promote bone development by cytokine and chemokine secretion, extracellular matrix production, and gene delivery in addition to the immunomodulation and promotion of angiogenesis [36, 37].

In addition, there was also a clinical study regarding vertebral osteomyelitis treatment by Kankare et al. They performed anterior decompression and reconstruction following decompression and posterolateral spondylodesis with transpedicular fixation. In this study, they used a vertebral body expander replacement device and bioactive glass S53P4 which has antibacterial activity, with bone bonding and osteoconductive ability. Three patients that presented with *Mycobacterium tuberculosis*, *Candida tropicalis*, and *S. aureus* vertebral infection were evaluated. Spinal fusion with full neurologic recovery and no further surgery needed was found in the follow-up. This might be due to the leaching and dissolution of alkali and alkaline ions from the bioactive glass resulting in maximum pH of 11 and increased osmotic pressure to deliver growth inhibitory effect and killing of the bacteria [5]. In the present study, our observation showed that new bone formation and ossification remained to occur with continuous infection of *M. tuberculosis*. However, the existed infection and inflammation may hamper and disrupt bone healing, formation, and regeneration. This was supported by our previous study showing increased calcium level and bone cell metabolism towards the lesion in which fewer osteocytes were contained in the defect area [2]. The use of MSCs and subsequent hydroxyapatite scaffold utilization were expected to help bone formation in the cases of large and infected defects, considering its anti-inflammatory and immunomodulatory activity suppressing the inflammatory process and immune responses towards *M. tuberculosis* infection.

UC-MSCs may be a promising alternative for large vertebral bone defects. However, our study is limited by the small sample that is eligible for this study. We only found 3 patients that are suitable to undergo the therapy. The recruitment process of the patients is limited by the high cost of the therapy and patients having concerns and not willing to undergo stem cell therapy. We also realized that our study lacks a control group to be compared to our therapy group. The data evaluated in our study is also from a single institution; the characteristics of the patients and study results may be specific to our institution only. Nevertheless, the present study can serve as the foundation for further therapy for VBD using UC-MSCs combined with HA granules in humans. This becomes the novelty of our study that yields good results when compared to today's gold standard using BM-MSCs. However, future studies with a larger sample and proper control may be needed.

## 5. Conclusion

Umbilical cord mesenchymal stem cells combined with hydroxyapatite-based scaffold utilization represent a prospective alternative therapy for bone formation and regeneration in the treatment of vertebral bone defect due to spondylitis tuberculosis. This study shows significant new bone formation in the defect area in line with the increase of ALP level and significant improvements in the term of SF-36 score after therapy. Further clinical investigations and assessments with a larger study size and appropriate control would be necessary to evaluate the quality, safety, and efficacy of this new alternative substitute.

## Figures and Tables

**Table 1:** Patient characteristics.

Case	Gender	Age	Affected region	Diagnosis	Previous surgery
1	Female	29	L1-L2 vertebrae	Implant failure with history of paraparesis due to compression fracture of L1-L2 vertebrae due to spondylitis tuberculosis Frankel D	Debridement, decompression, and posterior stabilization of L1-L2 vertebrae
2	Male	27	L3-L4 vertebrae	Back pain and leg pain due to spondylitis TB of L3-L4 with paravertebral abscess Frankel E	—
3	Female	30	T8-L1 vertebrae	Implant failure with exposed implant of T8-L1 due to spondylitis TB Frankel C	Debridement, decompression, and posterior stabilization of T8-L1 vertebrae

**Table 2:** The effect of UC-MSC implantation for vertebral bone defect due to spondylitis tuberculosis.

Case	Pre-op		1 month			3 months				6 months			
	ALP	SF-36	X-ray	ALP	SF-36	X-ray	CT scan	ALP	SF-36	X-ray	CT scan	ALP	SF-36
1	40 U/L	850	T1	45 U/L	1040	T3	B2	95 U/L	1780	T3	B2	79 U/L	1985
2	46 U/L	900	T0	58 U/L	1090	T2	B3	106 U/L	2800	T3	B2	77 U/L	2995
3	38 U/L	460	T0	42 U/L	690	T2	B2	90 U/L	1880	T4	B1	85 U/L	2830
Mean ± SD	41.3 ± 4.16 U/L	736.67 ± 240.90		48.33 ± 8.50 U/L	940 ± 217.94			97 ± 8.19 U/L	2340 ± 650.54			90.33 ± 4.16 U/L	2912.5 ± 116.67

## Data Availability

The data used to support the findings of this study are available from the corresponding author upon request.
